# Supplementary material for: A substitutional quantum defect in WS2 discovered by high-throughput computational screening and fabricated by site-selective STM manipulation
Source: Nat Commun. 2024 Apr 26;15:3556. doi: 10.1038/s41467-024-47876-3 (PMC11519662; doi:10.1038/s41467-024-47876-3)
Supplement: Supplementary file 1 — Supplementary Information [file 41467_2024_47876_MOESM1_ESM.pdf]

Supplementary Information for
A substitutional quantum defect in WS_2 discovered by high-throughput computational screening and fabricated by site-selective STM manipulation

John C. Thomas[1,2,3*†], Wei Chen[4†], Yihuang Xiong[3†], Bradford A. Barker[5], Junze Zhou[1], Weiru Chen[3], Antonio Rossi[1,2,6], Nolan Kelly[5], Zhuohang Yu[7,8], Da Zhou[9], Shalini Kumari[7,8], Edward S. Barnard[1], Joshua A. Robinson[7,8,9,10], Mauricio Terrones[7,8,9,10], Adam Schwartzberg[1], D. Frank Ogletree[1], Eli Rotenberg[6], Marcus M. Noack[11], Sinéad Griffin[1,2], Archana Raja[1,2], David A. Strubbe[5], Gian-Marco Rignanese[4], Alexander Weber-Bargioni[1,2*], and Geoffroy Hautier[3*]

[1]*Molecular Foundry, Lawrence Berkeley National Laboratory, Berkeley, CA 94720, United States of America*
[2]*Materials Sciences Division, Lawrence Berkeley National Laboratory, Berkeley, CA, United States of America*
[3]*Thayer School of Engineering, Dartmouth College, Hanover, NH 03755, USA*
[4]*Institute of Condensed Matter and Nanoscicence, Université catholique de Louvain, Louvain-la-Neuve 1348, Belgium*
[5]*Department of Physics, University of California, Merced, Merced, CA 95343, USA*
[6]*Advanced Light Source, Lawrence Berkeley National Laboratory, Berkeley, CA 94720, United States of America*
[7]*Department of Materials Science and Engineering, The Pennsylvania State University, University Park, PA 16082 United States of America*
[8]*Center for Two-Dimensional and Layered Materials, The Pennsylvania State University, University Park, PA, 16802 United States of America*
[9]*Department of Physics, The Pennsylvania State University, University Park, PA, 16802 United States of America*
[10]*Department of Chemistry, The Pennsylvania State University, University Park, PA, 16802 United States of America*
[11]*Applied Mathematics and Computational Research Division, Lawrence Berkeley National Laboratory, Berkeley, CA 94720, United States of America*
**jthomas@lbl.gov, afweber-bargioni@lbl.gov, geoffroy.hautier@dartmouth.edu*
[†]*These authors contributed equally.*

SUPPLEMENTARY NOTES

1| Autonomous Experimentation

A Gaussian process (GP) model can be defined for a given dataset, $D = \{x_i, y_i\}$, which takes into account $y(x) = f(x) + \varepsilon(x)$, where x are the positions in some input or parameter space, y is the associated noisy function evaluation, and $\varepsilon(x)$ represents the noise term. The variance-covariance matrix Σ of the prior Gaussian probability distribution is defined by Matérn kernel functions $k(x_i, x_j; \phi)$, where ϕ is the

set of hyperparameters found by maximizing the marginal log-likelihood of the data[1]. A predictive mean and variance can then be defined given a Gaussian probability distribution with a set of optimized hyperparameters, which can be further used to find the next optimal point measurements in the GP-driven data acquisition loop. For the results presented, an acquisition function that collects points to reduce uncertainty and improve the statistical model (exploration mode) was used. Drift was corrected during the autonomous experiment, where *x*-*y* offsets were calculated after each spectral loop and applied to the dataset.

2| 1D Convolutional Neural Network

Spectra for WS_2 and V_S were taken from Thomas et al. and used during training[1]. The one-dimensional convolutional neural network (CNN) chosen makes use of two convolution layers, one dropout layer, and one fully connected linear layer. We use an 80/20 train/validation split ratio on 394 individually and separately acquired scanning tunneling spectra, consisting of 45 Co_S, 158 V_S, and 191 WS_2 spectra. Validation data is further split (60/40 ratio) for a portion to be used during training, which yields an estimate of the model's skill, and a test set used on unbiased data after training. The softmax of the trained model is then used after training to obtain point STS class probabilities. All convolutional layers make use of a 1×3 kernel to compute the sliding dot product and produce spectral feature maps at each layer (stride 1, padding 1). This is followed by batch normalization, a rectified linear unit activation, and a maxpooling layer. The Adam algorithm[2] with a learning rate of 10^{-4} and computed cross-entropy loss for optimization are used during training to automatically identify spectral features. Spectra for WS_2, V_S, and Co_S that are unseen by the trained model are used for test data. The CNN architecture chosen uses shared weights to reduce the number of trainable parameters and extract spectral features on the pixel level.

SUPPLEMENTARY TABLE

Defect Database:

Defect	Total spin	ΔKS (eV)	TDM (Debye)
Br_W^0	1/2	0.854	5.79
Sc_S^0	1/2	0.8	3.01
Sb_W^-	0	1.037	6.33
Rb_W^-	1	0.825	10.01
Te_W^-	1/2	0.778	8.03
S_W^0	0	0.939	10.77
P_W^-	0	1.134	6.13
Ir_W^+	0	0.84	5.17
As_W^-	0	0.941	7.74
Pb_W^{-2}	0	1.035	6.95
C_W^{-2}	0	1.093	8.29
K_W^-	1	0.877	9.67
Ca_W^0	1	0.755	10.42
Ca_W^{-2}	0	0.794	9.89
Mg_S^+	1/2	0.786	4.23
N_W^-	0	1.08	9.56
Ru_W^0	0	0.937	3.97
Ru_W^+	1/2	0.824	3.06
Co_S^0	1/2	1.29	6.41
Bi_W^-	0	0.838	8.8
W_S^+	1/2	0.968	3.9
Vac_W^{-2}	1	0.76	7.97
Rh_W^-	0	0.788	7.45
Os_W^0	0	1.04	3.42
Fe_S^0	1	1.184	4.93
Sr_W^0	1	0.754	11.04
Sr_W^{-2}	0	0.78	10.68
Na_W^-	1	0.802	9.02
Zn_S^0	1	1.11	3.6
Ge_S^-	1/2	0.838	7.02
Ti_S^0	0	1.843	4.33
Li_S^0	1/2	0.764	3.8

Supplementary Table 1: All the thermodynamically stable two-level defect candidates that show transition dipole moment (TDM) larger than 2.5 D and Kohn-Sham energy difference (ΔKS) larger than 750 meV are summarized in the table below. The ΔKS is computed at single-shot PBE0 level using an α of 0.07, as detailed in the main text.

Effect of spin-orbit coupling (SOC) on Co_S defect levels:

	occupation	no SOC	SOC
$d_{x^2-y^2}$	0	−4.06	−4.06
d_{z^2}	1	−5.42	−5.40
$d_{xy}+d_{xz}$	1	−5.56	−5.53

Supplementary Table 2: Eigenvalues (in eV) of defect levels associated with the Co-3d states for the neutral Co_S^0 defect calculated within collinear (no SOC) and noncollinear spin-polarizations (with SOC). All energies are referred to the vacuum level.

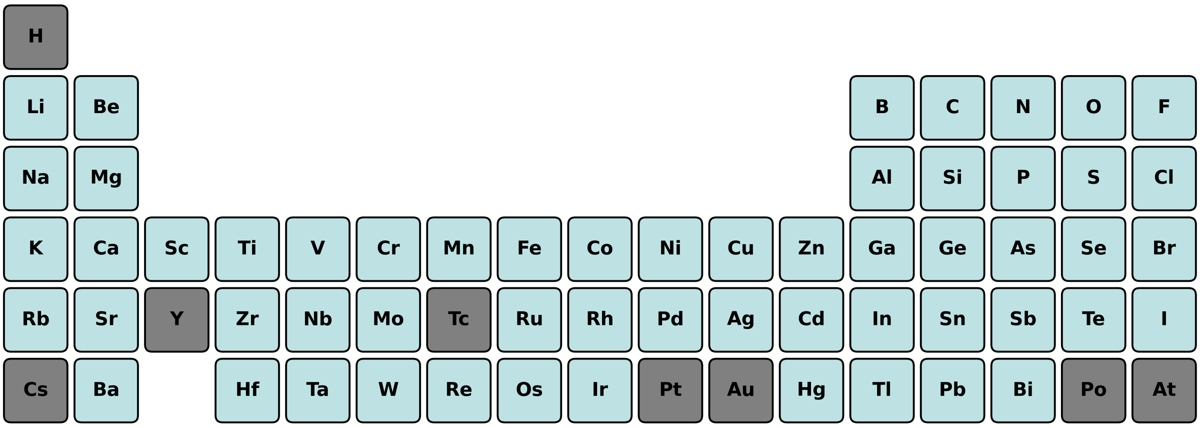

Supplementary Fig. 1: In this work, we considered 57 elements for constructing a quantum defect database in WS_2. Our selection covers the majority of the elements, excluding the rare-earth elements and noble gases. The elements that were not considered are colored in gray.

a

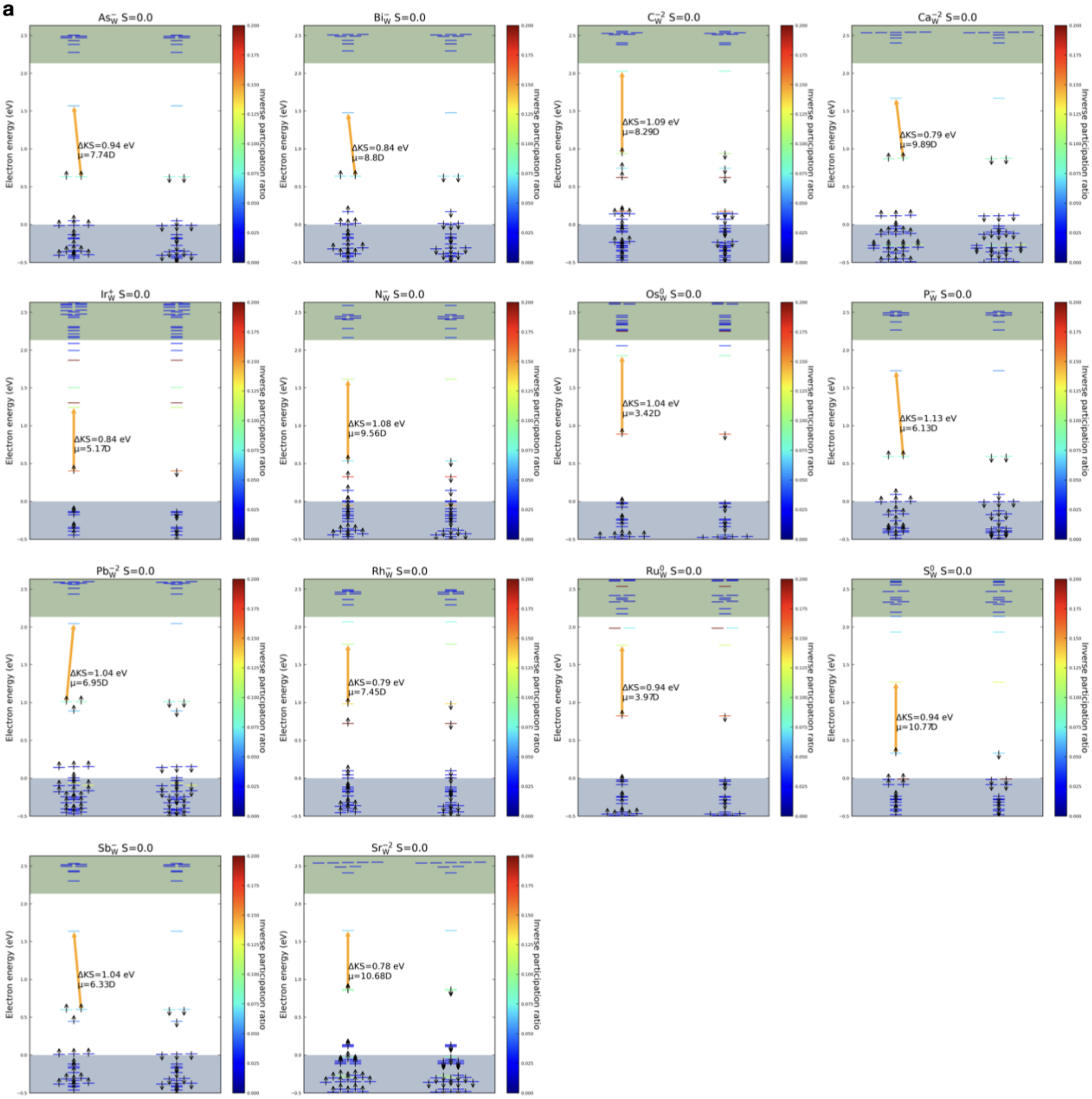

As$_W^-$ S=0.0
ΔKS=0.94 eV
μ=7.74D
Bi$_W^-$ S=0.0
ΔKS=0.84 eV
μ=8.8D
C$_W^{-2}$ S=0.0
ΔKS=1.09 eV
μ=8.29D
Ca$_W^{-2}$ S=0.0
ΔKS=0.79 eV
μ=9.89D
Ir$_W^+$ S=0.0
ΔKS=0.84 eV
μ=5.17D
N$_W^-$ S=0.0
ΔKS=1.08 eV
μ=9.56D
Os$_W^0$ S=0.0
ΔKS=1.04 eV
μ=3.42D
P$_W^-$ S=0.0
ΔKS=1.13 eV
μ=6.13D
Pb$_W^{-2}$ S=0.0
ΔKS=1.04 eV
μ=6.95D
Rh$_W^-$ S=0.0
ΔKS=0.79 eV
μ=7.45D
Ru$_W^0$ S=0.0
ΔKS=0.94 eV
μ=3.97D
S$_W^0$ S=0.0
ΔKS=0.94 eV
μ=10.77D
Sb$_W^-$ S=0.0
ΔKS=1.04 eV
μ=6.33D
Sr$_W^{-2}$ S=0.0
ΔKS=0.78 eV
μ=10.68D
Electron energy (eV)
Inverse participation ratio

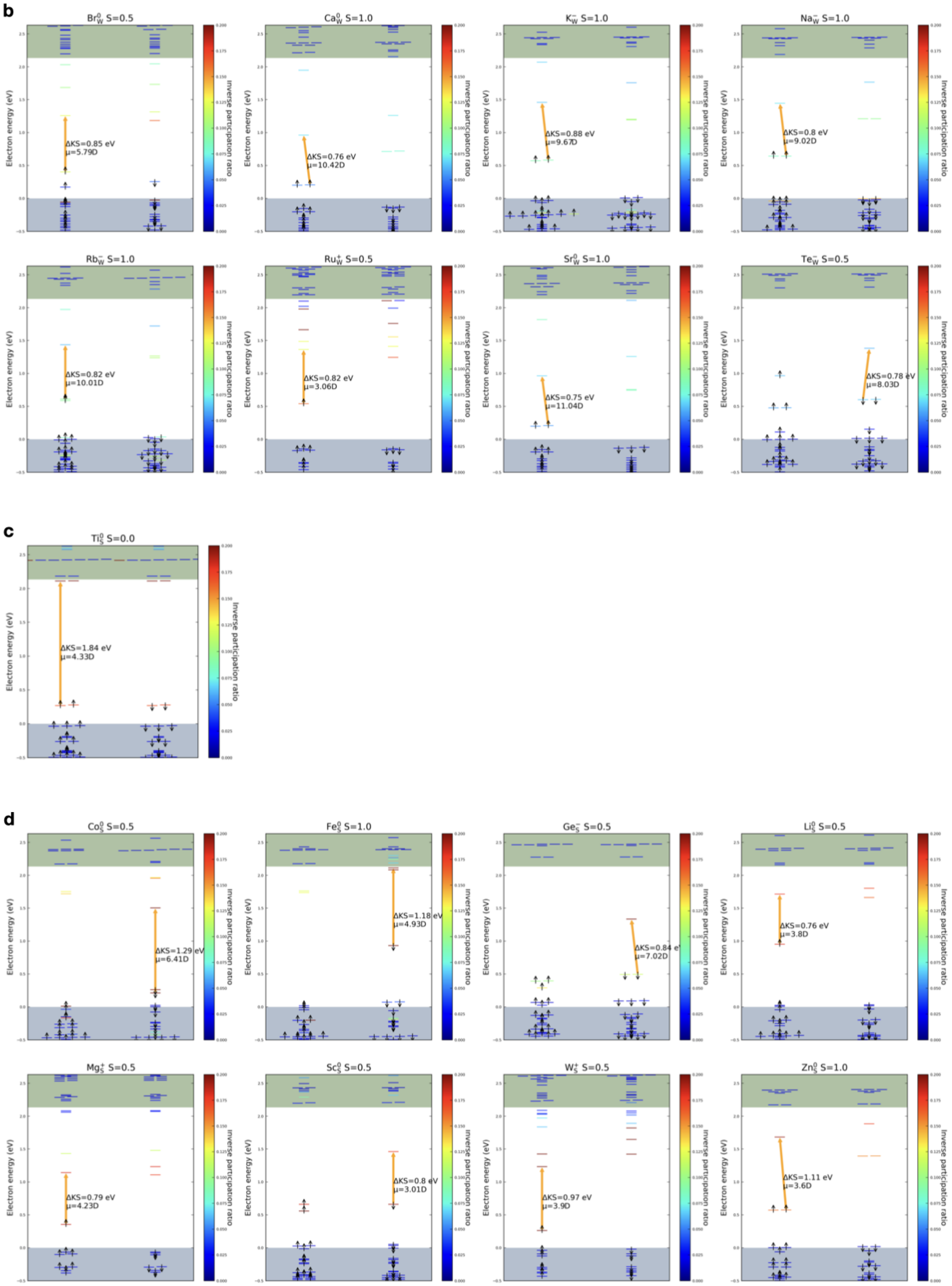

Supplementary Fig. 2: The single-shot PBE0 single-particle defect level diagrams of the screened candidates that have ΔKS > 750 meV and TDM larger than 2.5 D. The two levels that are involved in the transition are highlighted using the arrows, and the localization IPR represented by the color bar. These candidates are grouped into: **a** M_W defects with singlet ground states, **b** M_W defects with nonsinglet ground states, **c** M_S defects with singlet ground states, and **d** M_S defects with nonsinglet ground states.

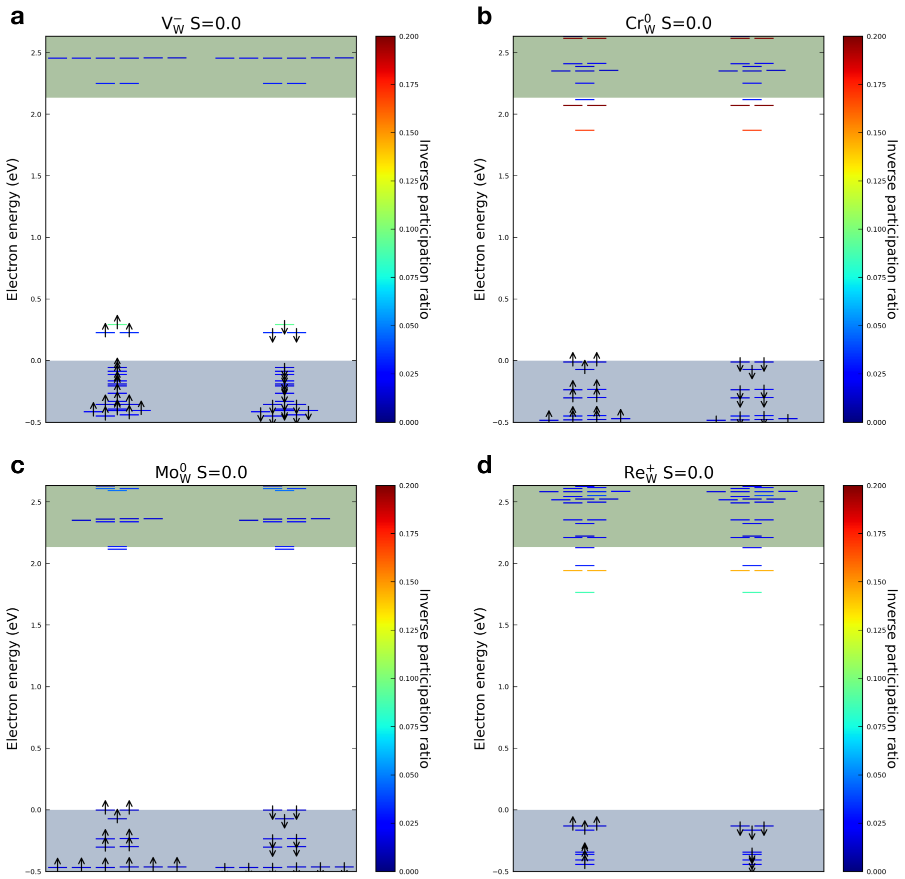

Supplementary Fig. 3: Single-shot PBE0 defect level diagrams of **a** V_W^-, **b** Cr_W^0, **c** Mo_W^0,and **d** Re_W^+ in monolayer WS_2.

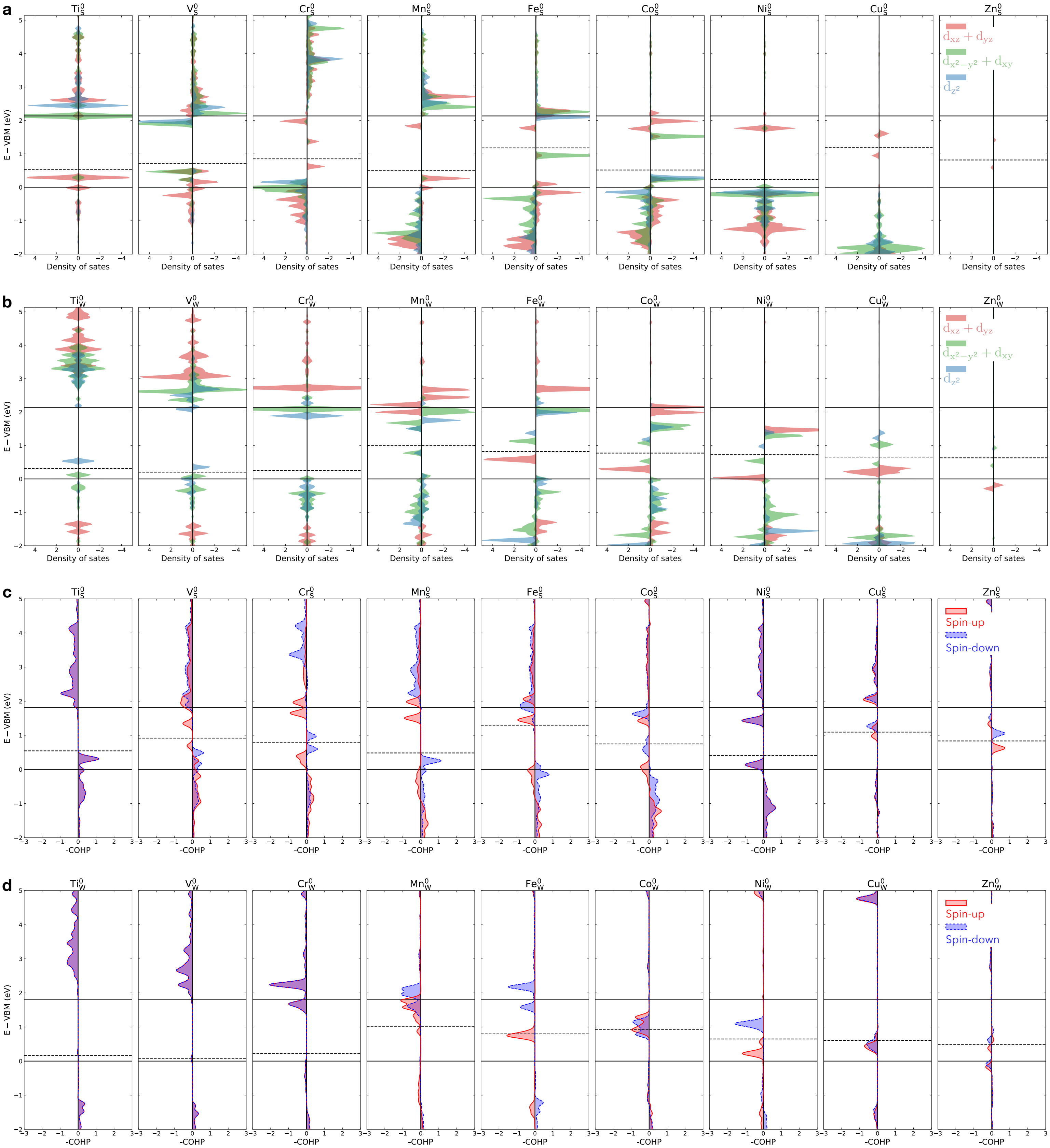

Supplementary Fig. 4: **Partial density of states and crystal orbital Hamilton population (COHP) analysis of 3*d* transition metal defects in WS_2.** Projected density of states of the transition metals for **a** substitution on S and **b** substitution on W. The shown density of states are computed at single-shot PBE0 level. The COHP of 3*d* defects substitution on **a** S and **b** W are evaluated using PBE wave functions. The Fermi level is shown with a dashed horizontal line and band edges are shown with solid horizontal lines.

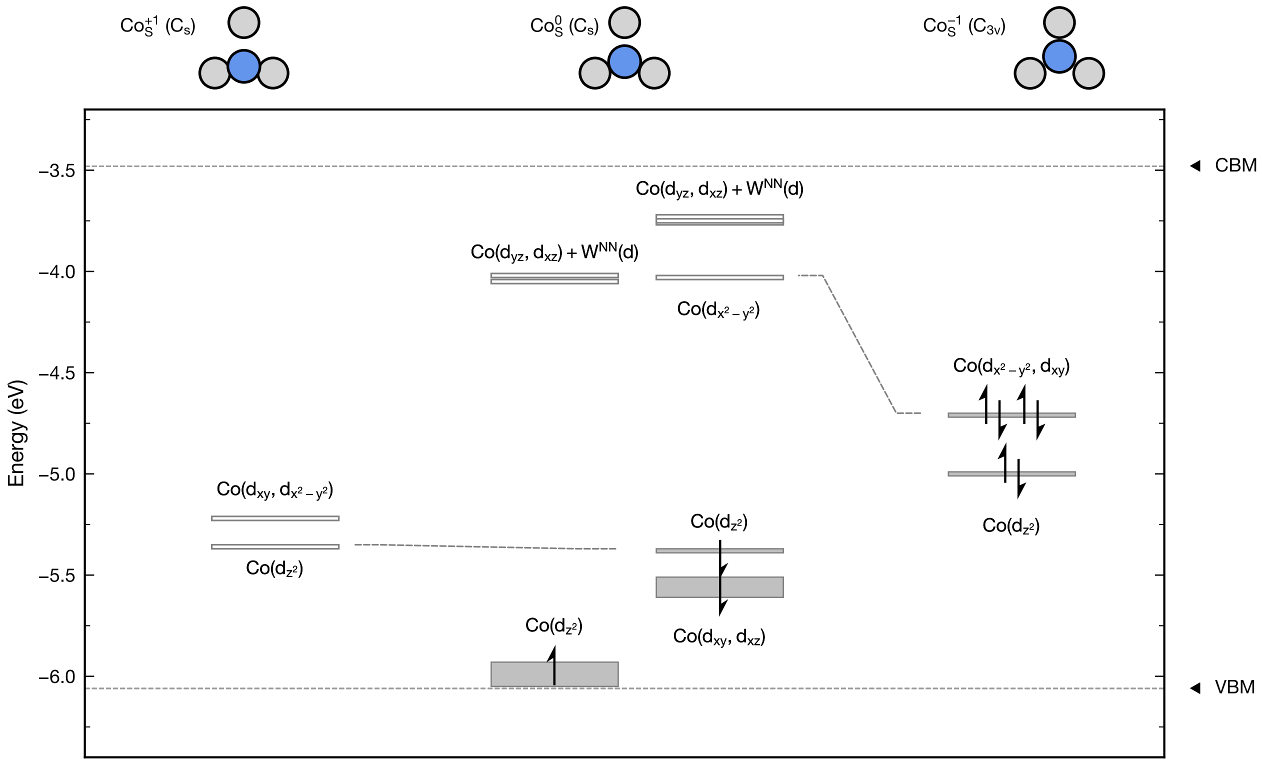

Supplementary Fig. 5: **Co$_S$ defect energy levels**. Localized defect states are shown in the +1, 0, and −1 charge states. Resonant states within the valence band and conduction band manifolds are not depicted. The associated single-particle levels are indicated by the horizontal bars (closed for occupied and open for unoccupied states). The dashed lines connect the active orbitals responsible for charging/discharging between two charge states. The band-edge positions indicated in the plot refer to the ones for the pristine WS_2, which are obtained from PBE0 calculations by admixing 22% of Fock exchange. SOC is only applied to the band edges.

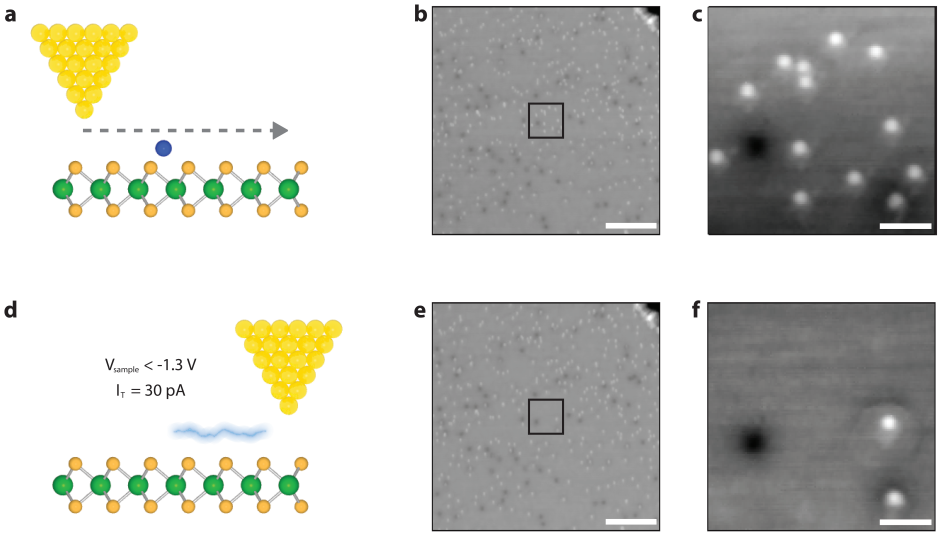

Supplementary Fig. 6: **Tip-induced evaporation/diffusion.** **a** An atomically sharp tip is rastered across a adsorbed Co defect site. **b**, **c** Scanning tunneling micrographs depicting pristine WS_2 with submonolayer Co atoms adsorbed before local diffusion/evaporation events (I_{tunnel} = 30 pA, V_{sample} = 1.2 V). Scale bars, 20 nm and 3 nm, respectively. **d** After scanning the local region in **c** at excitation voltage of -1.4 V, the majority of atoms are evaporated (or diffused to a defect capable of absorption (i.e., V_S)). **e**, **f** Scanning tunneling images of the same large-scale and excited region, where the majority of Co atoms remain that have not been exposed to tunneling-induced motion (I_{tunnel} = 30 pA, V_{sample} = 1.2 V). Scale bars, 20 nm and 3 nm. Predicted stable sites of adsorbed Co before tip-induced excitation include above a W site, S site, and a hollow site, where any remaining Co adatoms, after a tip-induced event, are expected to remain in a more stable W site[3,4].

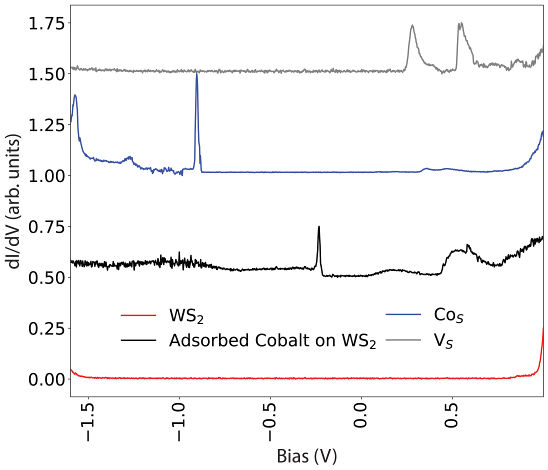

Supplementary Fig. 7: **Point STS Comparison.** dI/dV spectra recorded on as-grown WS_2 (red), adsorbed Co atop WS_2 (black), the Co_S defect (blue), and a typical V_S (gray) are presented above ($V_{modulation}$ = 5 mV). The as-measured energy gap of an adsorbed Co is 0.97 ± 0.27 eV and 2.0 ± 0.05 eV for Co_S. Both WS_2 and V_S recorded energy gaps and point spectra match values that have been reported in the literature[5].

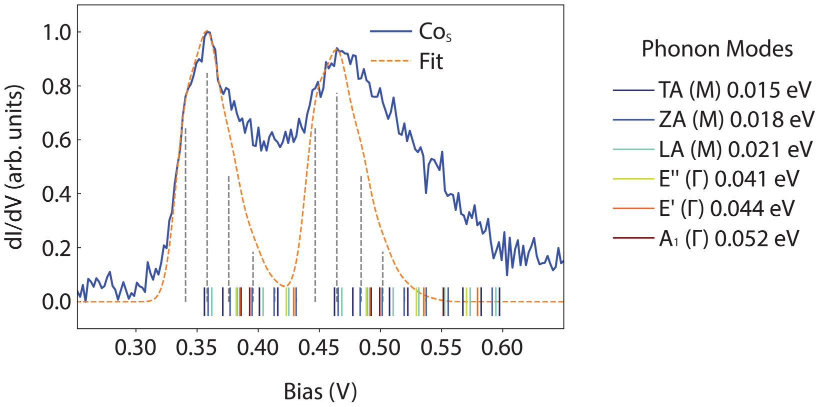

Supplementary Fig. 8: **Phonon Excitation Comparison.** The first and second peaks ($\hbar\omega_{eg}$) within acquired dI/dV are fitted according to the single-mode Franck-Condon model at both 0.36 eV and 0.47 eV. This can be fit as $\frac{dI}{dV}(V) = A\sum_{n=0}^{\infty} e^{-S}\frac{1}{n!}S^n\delta(eV - \hbar\omega_{eg} - n\hbar\omega_0)$, where A is an arbitrary scaling factor, S is the Huang-Rhys factor, $\hbar\omega_{eg}$ is the electronic excitation energy or zero-phonon line, and $\hbar\omega_0$ is the excited phonon mode. We use a Gaussian function with a full width at half maximum (Γ) to replace the δ function and account for broadening. Phonon modes were taken from literature values[6,7]. A Huang-Rhys factor of S = 1.3, a first excitation of $\hbar\omega_{eg}$ = 0.341 eV, a phonon mode of $\hbar\omega_0$ = 0.018 eV, and a broadening of Γ = 0.021 eV is estimated for the lower energy defect state, and S = 1.2, a first excitation of $\hbar\omega_{eg}$ = 0.447 eV, $\hbar\omega_0$ = 0.018 eV, and Γ = 0.021 eV is estimated for the next available unoccupied state with higher energy.

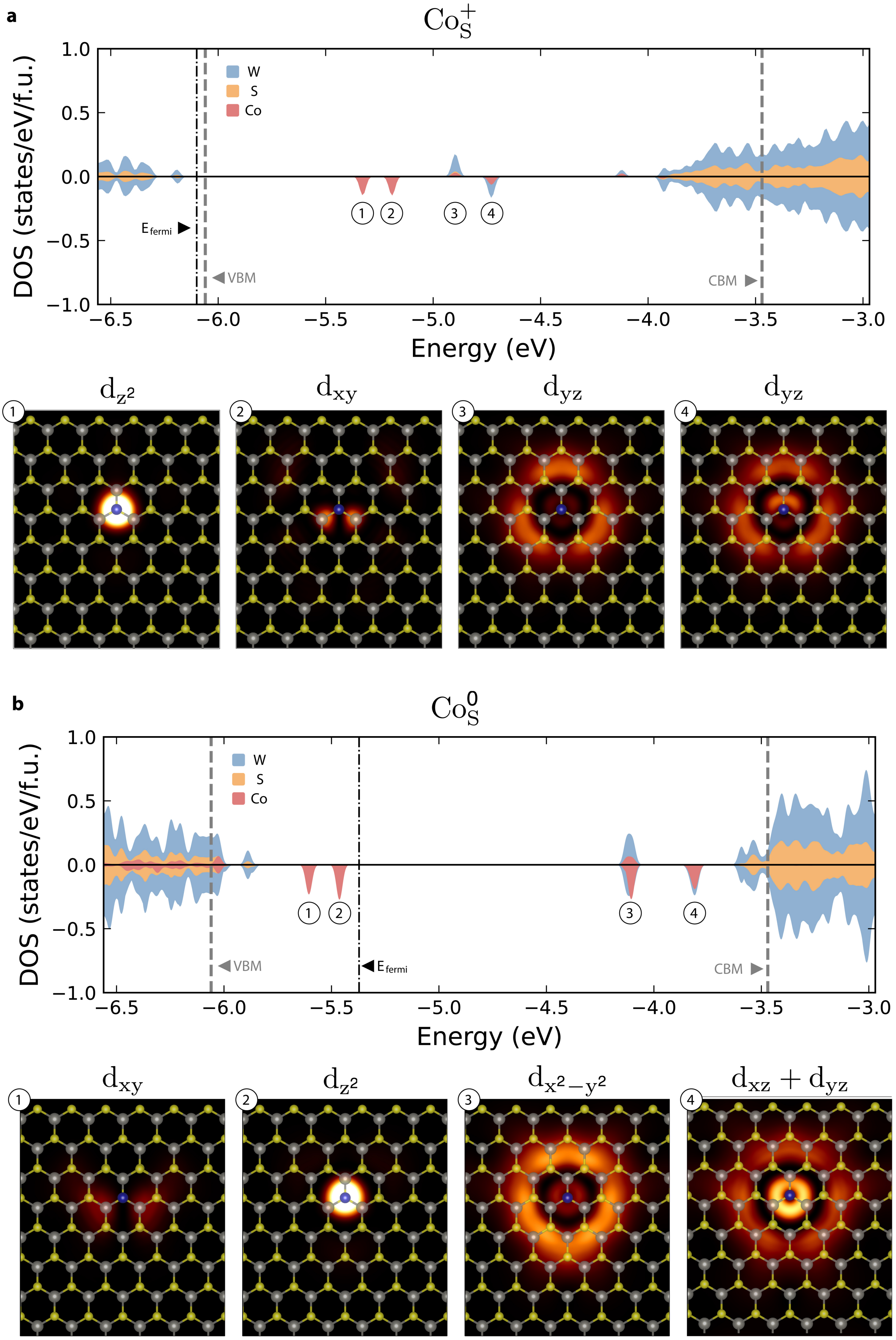
a
Co_S^+
DOS (states/eV/f.u.)
W
S
Co
E fermi
VBM
CBM
1.0
0.5
0.0
−0.5
−1.0
−6.5
−6.0
−5.5
−5.0
−4.5
−4.0
−3.5
−3.0
Energy (eV)
1 2 3 4
d_{z^2}
d_{xy}
d_{yz}
d_{yz}
b
Co_S^0
DOS (states/eV/f.u.)
W
S
Co
VBM
E fermi
CBM
Energy (eV)
d_{xy}
d_{z^2}
$d_{x^2-y^2}$
$d_{xz} + d_{yz}$

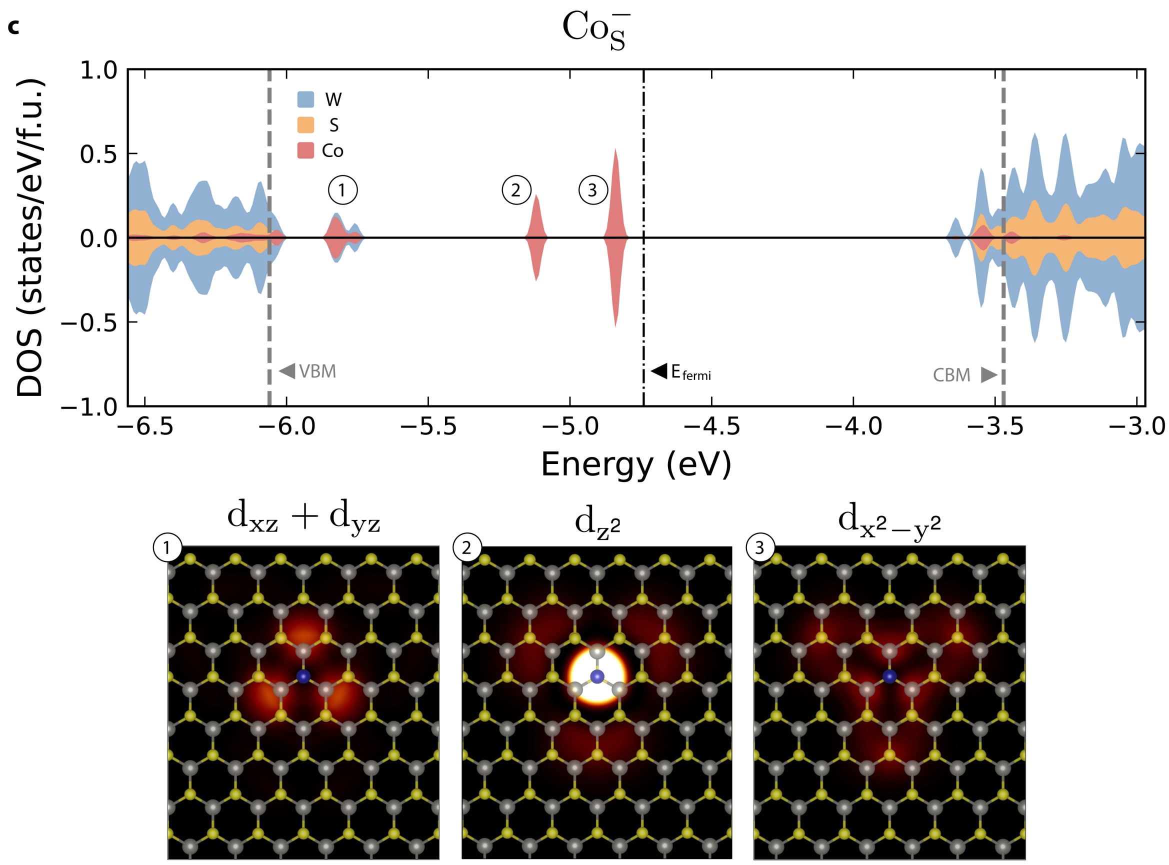

Supplementary Fig. 9: **Element-resolved density of states (DOS) of Co_S in WS_2**. We considered **a** +1, **b** neutral, and **c** -1 charge states. The Scanning Tunneling Spectroscopy results are simulated using PBE0 charge density and energy levels. Wavefunctions of atomic orbitals of Co that contribute most to each Co-related in-gap state are shown below the DOS.

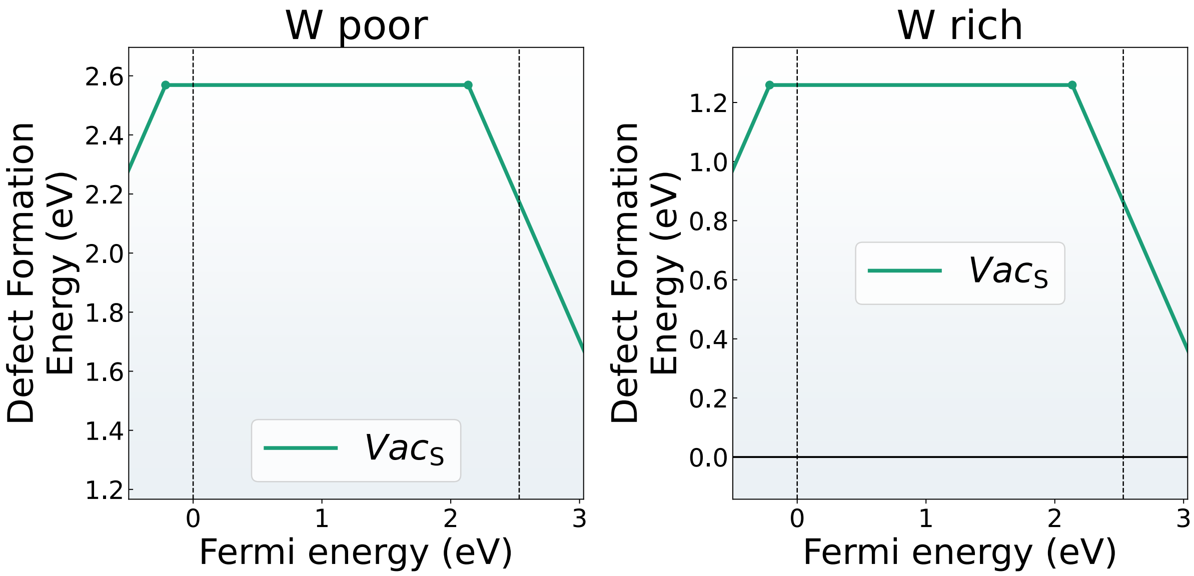

Supplementary Fig. 10: **Thermodynamic charge transition levels of S vacancy in WS_2.** The defect formation energies are evaluated under W-rich conditions ($\mu_S = -6.25$ eV, $\mu_W = -13.83$ eV) and W-poor conditions ($\mu_S = -4.94$ eV, $\mu_W = 16.45$ eV). The $0/-1$ charge transition level is at 2.14 eV with respect to VBM. The vertical dotted lines indicate the band edges.

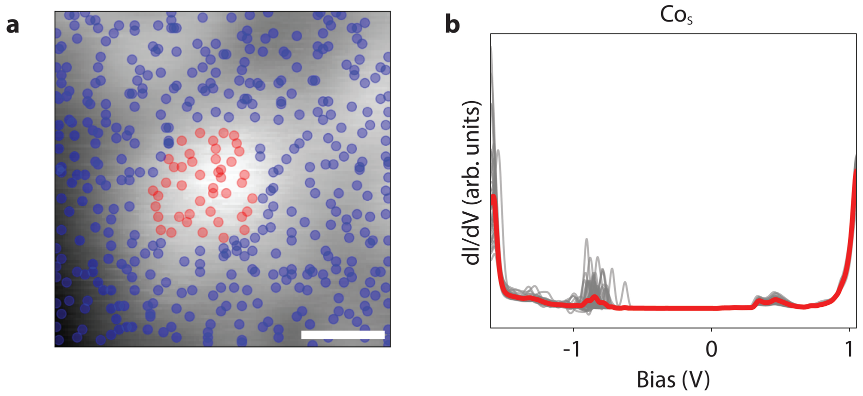

Supplementary Fig. 11: **Hyperspectral Data Collection. a** Co_S is identifiable by point bias spectroscopy followed by classification using a trained 1D-CNN, where image tracking can be performed on the defect of interest during an autonomous STS experiment. Outside spectra (either pristine WS_2 or V_S) are bucketed under a different classification (shown in blue). Acquired point STS locations are overlaid on acquired topography (I_{tunnel} = 30 pA, V_{sample} = 1.2 V). Scale bar, 0.5 nm. **b** Accumulated spectra over Co_S are shown with the mean spectrum that is colored by classification, where a charging peak is measured at -0.84 ± 0.06 eV.

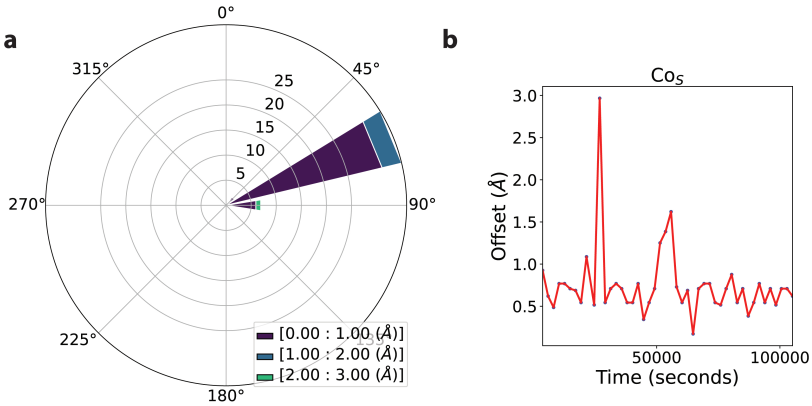

Supplementary Fig. 12: **Drift Correction.** Computed offsets during a Co$_S$ autonomous experiment is shown **a** in two dimensions and **b** the magnitude of the 2D vector as a function of time. Drift is multi-directional, but primarily near 70°. In order to correct for drift at each point, a drift rate was calculated between acquired images (Å/s) at every interval, and subsequently applied to each timestamped spectra.

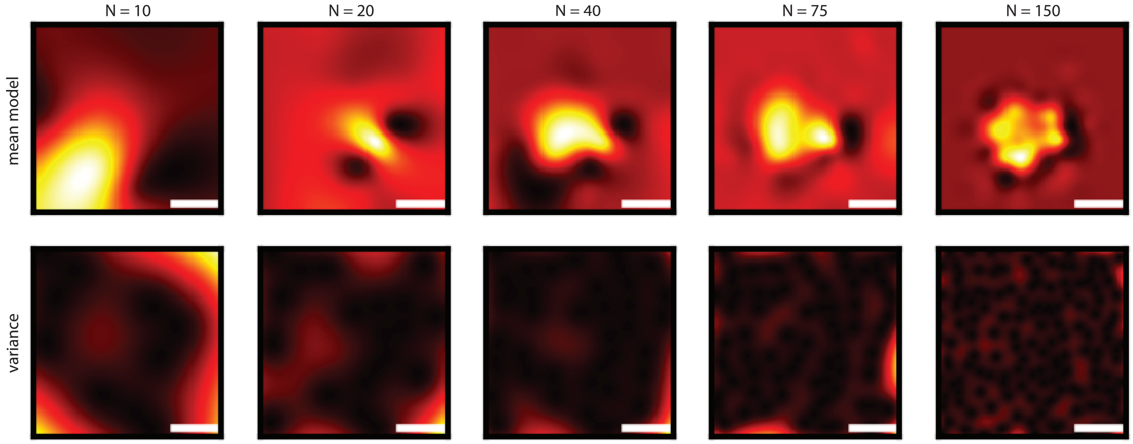

Supplementary Fig. 13: **Autonomous Experimentation.** A live experimental run within a range of 0.25 V to 0.65 V, which is chosen to highlight in-gap states for Co$_S$ below the conduction band and above E_F. Scale bars, 0.5 nm. Both the mean model function and variance function are shown at a given interval (N) as the experiment progresses in exploration mode.

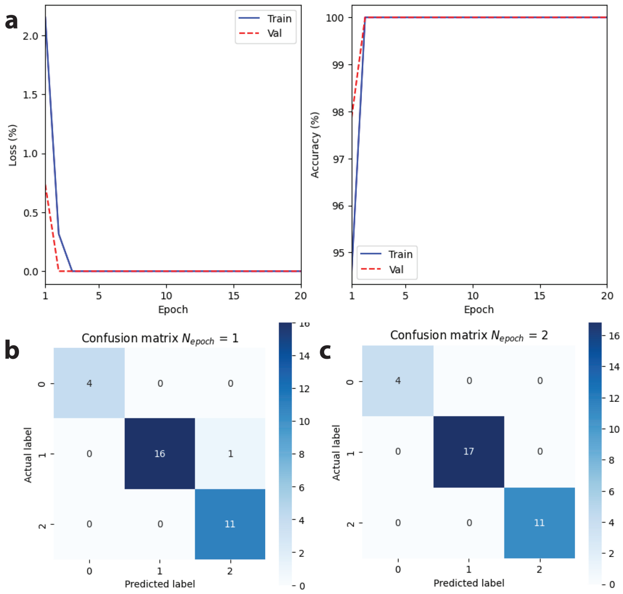

Supplementary Fig. 14: **1D Convolutional Neural Network Performance.** a) Accuracy and loss after 20 training epochs is shown on both training and validation datasets. b) Confusion matrices taken after classification on test data using the argmax value across class probabilities (yielded by the softmax). Training can be concluded after 2 epochs, where test data shows zero off-diagonal elements, loss is minimized, and accuracy is optimized.

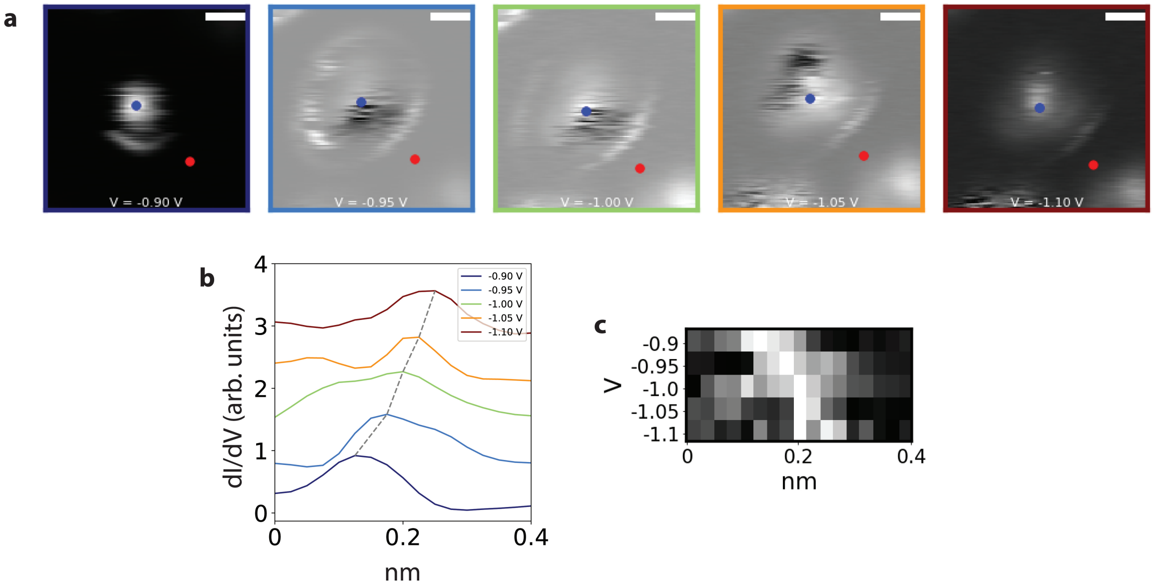

Supplementary Fig. 15: **Co$_S$ Charging Region.** **a** High resolution scanning tunneling differential conductance maps at -0.90 eV, -0.95 eV, -1.00 eV, -1.05 eV, and -1.10 eV ($V_{modulation}$ = 5 meV). Scale bars, 0.5 nm. Solving for the local maxima at the center of Co$_S$ and taking a same-size line scan from the center position to outside the charging region highlights the energetic shift as the bias is ramped to more negative values (shown spatially). Outside the ring, WS_2 remains neutral and, inside the ring, Co$_S$ is negatively charged at a given bias. **b** This is shown further as a compilation of linescans and a **c** compiled image as a function of distance and centered along the charging ring.

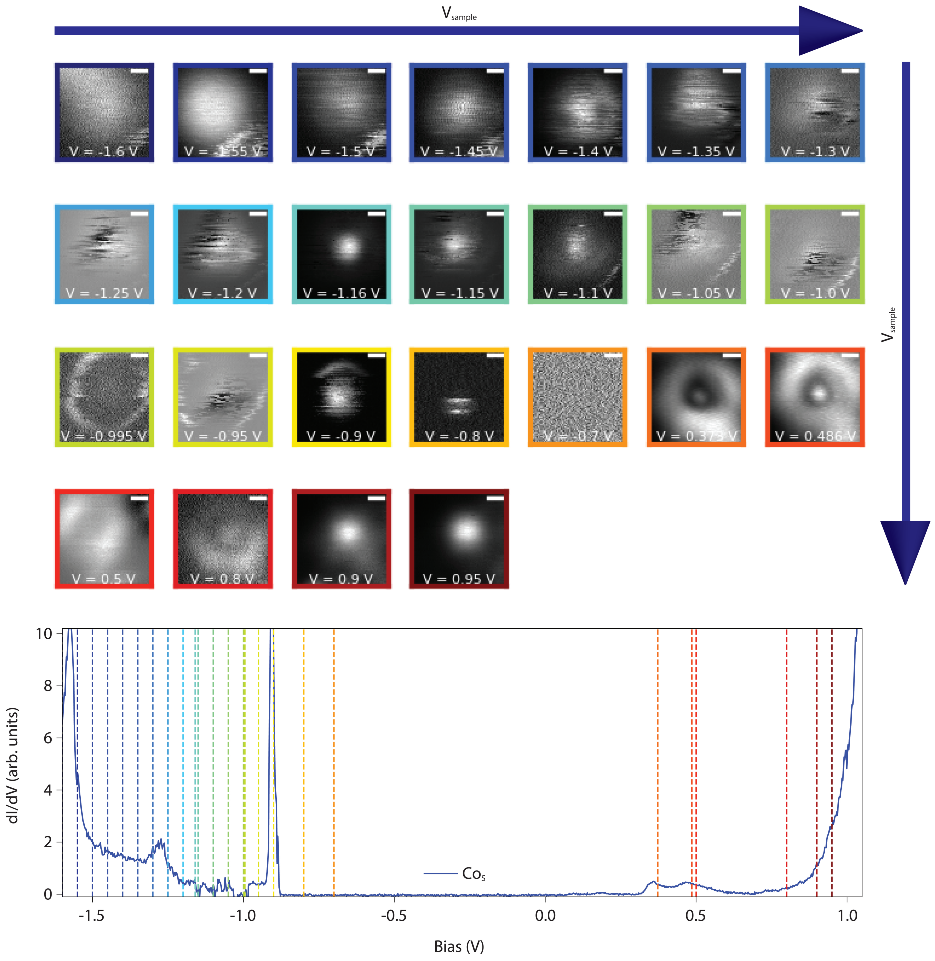

Supplementary Fig. 16: **Differential Conductance Mapping.** dI/dV images ($V_{modulation}$ = 5 meV) over the point STS ($V_{modulation}$ = 5 meV) region shown for a Co_S defect using a jet color scale, where the energy is ramped from near the VBM of WS_2 to -0.7 eV, from unoccupied peaks of interest, and then to below the CBM of WS_2. Scale bars, 0.25 nm. Orbitals of the as-formed Co_S and surrounding V_S are visualized as a function of bias voltage. Charging effects are only present in negative sample bias regimes.

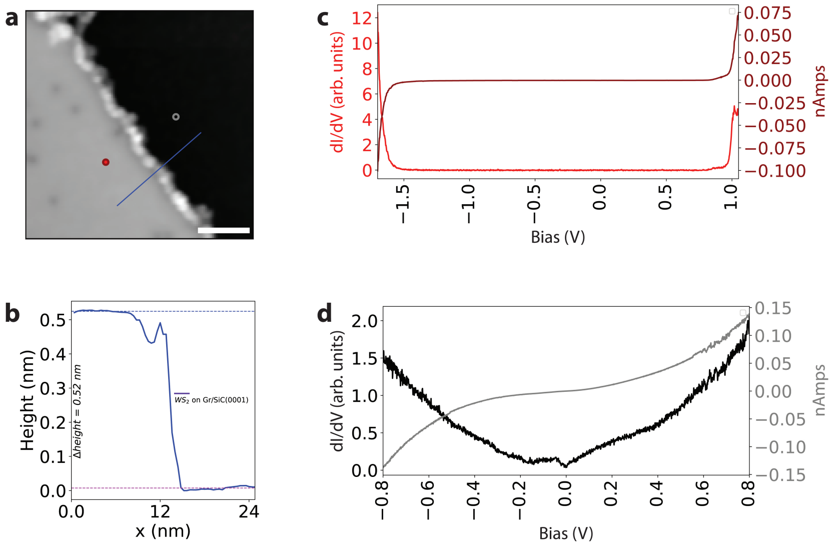

Supplementary Fig. 17: **Monolayer WS_2 Identification.** **a** Scanning tunneling image over a WS_2 monolayer edge resting on a graphene/SiC(0001) substrate (I_{tunnel} = 30 pA, V_{sample} = 1.2 V). Scale bar, 10 nm. **b** A height profile taken across the blue line depicted in **a**, where a height difference of ∼0.5 nm is measured. Regions are futher verified with scanning tunneling spectroscopy over both **c** as-grown WS_2 (red circle in **a**) and **d** the graphene/SiC substrate (gray circle in **a**) ($V_{modulation}$ = 5 mV, I_{set} = 150 pA). A band gap of 2.5 eV is measured for as-grown WS_2, and graphene exhibits expected canonical band structure.

Supplementary References

[1] Thomas, J. C. et al. Autonomous scanning probe microscopy investigations over WS_2 and Au{111}. *npj Comput. Mater.* **8**, 99 (2022).

[2] Kingma, D. P. & Ba, J. Adam: A method for stochastic optimization. Preprint available at https://arxiv.org/abs/1412.6980 (2014).

[3] Majd, Z. G., Taghizadeh, S. F., Amiri, P., & Vaseghi, B. Half-metallic properties of transition metals adsorbed on WS_2 monolayer: A first-principles study. *J. Magn. Magn. Mater.* **481**, 129 (2019).

[4] Xu, W. Electronic structures and magnetic properties of co-adsorbed monolayer WS_2. *J. Mater. Sci. Chem. Eng.* **4**, 32 (2016).

[5] Schuler, B. et al. Large spin-orbit splitting of deep in-gap defect states of engineered sulfur vacancies in monolayer WS_2. *Phys. Rev. Lett.* **123**, 076801 (2019).

[6] Molina-Sánchez, A. & Wirtz, L. Phonons in single-layer and few-layer MoS_2 and WS_2. *Phys. Rev. B* **84**, 155413 (2011).

[7] Molas, M. R., Nogajewski, K., Potemski, M., & Babiński, A. Raman scattering excitation spectroscopy of monolayer WS_2. *Sci. Rep.* **7**, 5036 (2017).
